# Machine learning-based prediction of gout flares during hospitalization in patients with upper gastrointestinal bleeding: a retrospective cohort study

**DOI:** 10.3389/fmed.2026.1807548

**Published:** 2026-06-10

**Authors:** Rongrong Chen, Sihan Hu, Shiyun Lu, Mengshi Chen

**Affiliations:** 1 Shengli Clinical Medical College of Fujian Medical University, Fuzhou, Fujian, China; 2 Department of Gastroenterology, Fuzhou University Affiliated Provincial Hospital, Fuzhou, Fujian, China; 3 School of Public Health, Guangxi Medical University, Nanning, China

**Keywords:** gout flare, hospitalization, machine learning, prediction model, random forest, upper gastrointestinal bleeding

## Abstract

**Background and aims:**

Acute gout flares pose a significant therapeutic challenge in hospitalized patients with upper gastrointestinal bleeding (UGIB) due to the contraindications of standard anti-inflammatory treatments. This study aimed to develop and validate a machine learning (ML) model to predict the risk of gout flares in this high-risk inpatient population.

**Methods:**

A retrospective cohort study was conducted on UGIB patients admitted to the Department of Gastroenterology, Provincial Hospital, Fujian Medical University. Five ML algorithms—Decision Tree (DT), Random Forest (RF), k-Nearest Neighbors (KNN), Naive Bayes (NB), and Extreme Gradient Boosting (XGBoost)—were trained and tested using routinely collected clinical and laboratory data at admission. Model performance was evaluated on an independent test set using the area under the receiver operating characteristic curve (AUC), accuracy, sensitivity, specificity, and decision curve analysis (DCA). SHapley Additive exPlanations (SHAP) were used to enhance model interpretability.

**Results:**

A total of 718 patients were included, with 158 (22.0%) experiencing a gout flare during hospitalization. The RF model exhibited the best predictive performance, achieving an AUC of 0.951 (95% CI: 0.923–0.979), accuracy of 0.901, sensitivity of 0.929, and specificity of 0.873 on the test set. DCA confirmed the clinical utility of all models. SHAP analysis identified six key predictors: serum uric acid (UA), creatinine (Cr), hemoglobin (HB), blood urea nitrogen (BUN), body mass index (BMI), and alcohol consumption history.

**Conclusion:**

This study successfully developed a robust ML model, with RF as the optimal algorithm, for accurately predicting inpatient gout flares in UGIB patients. This tool facilitates early identification of high-risk individuals, enabling targeted preventive strategies and enhancing clinical management.

## 1 Introduction

Upper gastrointestinal bleeding (UGIB) is a common clinical emergency often managed through fluid resuscitation, pharmacological interventions such as proton pump inhibitors (PPIs), and invasive procedures like endoscopic hemostasis (1). During acute stress, patients commonly experience significant metabolic and internal environmental disturbances (2). Gout, a crystal-induced arthritis linked to purine metabolism disorders and/or impaired uric acid (UA) excretion, has become a notable complication in UGIB patients during hospitalization (3). A clinical dilemma arises when first-line treatments for acute gout flares, such as nonsteroidal anti-inflammatory drugs (NSAIDs) and glucocorticoids, pose risks to the gastrointestinal mucosa. NSAIDs can compromise the mucosal barrier (4), while glucocorticoids increase the risk of rebleeding or ulcer perforation (5). Consequently, gout flares exacerbate patient discomfort, extend hospitalization (6), and may negatively affect the prognosis of the underlying condition. At present, there is a lack of effective risk-stratification tools to predict gout flares in hospitalized UGIB patients, hindering early detection and preventive intervention. Notably, recent research suggests that PPI use may be a contributing factor to gout flares in this population, highlighting the complexity of managing these coexisting conditions (7).

Machine learning (ML) methods offer significant advantages in clinical prediction modeling (8, 9). In contrast to traditional statistical techniques such as logistic regression, ML is more effective in managing high-dimensional data, variable collinearity, and nonlinear relationships, making it particularly suitable for complex clinical datasets (10, 11). Consequently, the development of an ML-based prediction model may enhance the accurate identification of high-risk patients for gout flares among those with UGIB, thereby optimizing clinical intervention strategies. Recent advancements have highlighted the superior performance of ML models over conventional methods in predicting outcomes related to gastrointestinal bleeding (12–14), reinforcing their applicability in this domain.

Current gout management guidelines, including the newly published International Clinical Practice Guideline of Chinese Medicine-Gout (SCM73-2024), primarily target general outpatient populations and lack specific recommendations for acute care settings complicated by contraindications such as UGIB (15). As such, there is an urgent need for risk stratification tools tailored to this unique inpatient population.

This study aims to develop and validate an ML model using retrospective clinical data to predict the risk of acute gout flares during hospitalization in UGIB patients. By analyzing clinical and laboratory parameters early after admission, key predictors will be identified, with the goal of providing data-driven support for early intervention, alleviating therapeutic conflicts, and ultimately improving patient outcomes.

## 2 Methods

### 2.1 Study design and population

This single-center, retrospective cohort study screened consecutive adult patients (aged ≥18 years) admitted with a primary diagnosis of UGIB to the Department of Gastroenterology at the Fuzhou University Affiliated Provincial Hospital between 2012 and 2025. UGIB diagnosis was confirmed through clinical presentation (e.g., hematemesis, melena) and endoscopic evaluation. Patients with a known history of gout or those who experienced a gout flare within the first 24 h of admission were excluded to ensure that the prediction was based solely on admission status for incident flares during hospitalization. Please refer to Supplementary Figure 1 for the inclusion and exclusion process. The study protocol was approved by the Hospital’s Institutional Review Board (IRB) [K2025-10-004], which waived the requirement for informed consent due to the retrospective nature of the analysis.

### 2.2 Data collection and predictor variables

Demographic data, medical history (including smoking and alcohol use), vital signs, and comprehensive laboratory results obtained within the first 24 h of admission were extracted from the electronic medical records. Predictor variables included: age, gender, body mass index (BMI), smoking history, fasting plasma glucose (FPG), lipid profiles (low-density lipoprotein cholesterol [LDL-C], high-density lipoprotein cholesterol [HDL-C]), liver enzymes (AST, ALT), renal function markers (serum creatinine [Scr]. blood urea nitrogen [BUN], serum UA, phosphorus [P]), and hemoglobin (HB). The primary outcome was the occurrence of an acute gout flare during the index hospitalization, diagnosed by the treating rheumatology or internal medicine team based on typical clinical presentation (acute monoarticular arthritis) and/or response to colchicine, in accordance with established clinical criteria.

### 2.3 Statistical analysis

#### 2.3.1 Descriptive analyses

In descriptive analyses, continuous variables were summarized as medians with interquartile ranges (IQR) due to skewed distributions. Categorical variables were expressed as counts and percentages. Differences in variable distributions between the gout and non-gout groups were compared using the rank sum test for continuous variables and the chi-square test for categorical variables (16).

#### 2.3.2 Construction of predictive models

An integrated ML analytical framework was developed to systematically examine the associations between all variables and gout incidence. The complete dataset was initially split into a training set (70%) for model development and a test set (30%) for validation using random sampling. A comparative analysis was conducted to assess the distributional differences between the two subsets to confirm the randomness of the split and ensure their comparability. Continuous variables were standardized to eliminate the influence of varying scales on geometric distance metrics, a critical preprocessing step for scale-sensitive models (17).

For predictive modeling, five distinct ML models were implemented in parallel: Decision Tree (DT), Random Forest (RF), Naive Bayes (NB), k-Nearest Neighbors (KNN), and Extreme Gradient Boosting (XGBoost). Hyperparameter tuning for each model was performed using a coordinated strategy of 10-fold cross-validation combined with an exhaustive grid search across parameter ranges (18). The optimally tuned model was then retrained on the entire training set, and its performance was rigorously evaluated on the test set. A comprehensive set of metrics was employed for evaluation, including the area under the receiver operating characteristic (ROC) curve (AUC) with its confidence interval (CI), accuracy, sensitivity, specificity, positive predictive value (PPV), negative predictive value (NPV), kappa value, and F1-score, with AUC serving as the primary evaluation criterion. Then, a simple multivariable logistic regression model was fitted using the training set, and its predictive performance was also subsequently evaluated on the independent test set. Decision curve analysis was also used to quantitatively assess the clinical utility of the prediction models by estimating the net benefit across various clinically relevant threshold probabilities (19). Additionally, calibration curve analysis was applied to examine the agreement between the model’s predicted probabilities and actual outcomes. The model demonstrating superior discriminatory performance was selected for further analysis.

To enhance the interpretability of the ML models, SHapley Additive exPlanations (SHAP) analysis, a technique based on cooperative game theory, was employed. This method quantifies the marginal contribution of each feature to individual predictions, providing a global ranking of feature importance and elucidating the direction and magnitude of each feature’s impact on gout risk, thus offering clinically actionable insights into the model’s decision-making process (20).

Finally, the prediction model was deployed as an interactive Shiny web application, enabling online access and visualization. All statistical analyses were performed using R software (version 4.5.0), with statistical significance defined as a two-tailed *p* < 0.05.

## 3 Results

### 3.1 Characteristics of study participants

Table 1 summarizes the baseline characteristics of the study population. The analysis revealed statistically significant differences between the gout and non-gout groups in the following variables: gender, smoking history, BMI, FPG, HDL-C, Scr, UA, BUN, and HB (all *p* < 0.05), indicating significant associations between these factors and gout status. However, no statistically significant differences were observed between the groups for age, LDL-C, AST, ALT, and P.

**Table 1:** Characteristics of participants grouped by gout status.

Variables	Overall	Non-gout group	Gout group	*p*
*n*	718	560	158	
Gender, *n* (%)				<0.001
Male	563 (78.4)	413 (73.8)	150 (94.9)	
Female	155 (21.6)	147 (26.2)	8 (5.1)	
Age, [median (IQR), years]	61.000 (47.000, 73.000)	61.000 (47.000, 73.000)	59.000 (43.000, 72.000)	0.337
Smoking history, *n* (%)				<0.001
No	558 (77.7)	474 (84.6)	84 (53.2)	
Yes	160 (22.3)	86 (15.4)	74 (46.8)	
Drinking history, *n* (%)				<0.001
No	553 (77.0)	478 (85.4)	75 (47.5)	
Yes	165 (23.0)	82 (14.6)	83 (52.5)	
BMI [median (IQR), kg/m2]	21.670 (20.450, 24.220)	21.245 (20.310, 22.835)	24.735 (23.560, 26.855)	<0.001
FPG [median (IQR), mmol/L]	6.390 (5.060, 8.418)	6.165 (4.900, 8.342)	6.975 (5.680, 9.092)	<0.001
LDL-C [median (IQR), mmol/L]	1.965 (1.460, 2.527)	1.930 (1.440, 2.502)	2.120 (1.460, 2.650)	0.242
HDL-C [median (IQR), mmol/L]	0.860 (0.670, 1.060)	0.875 (0.680, 1.070)	0.760 (0.580, 1.020)	0.002
AST [median (IQR), U/L]	19.000 (15.000, 28.550)	19.450 (15.000, 31.400)	18.000 (15.000, 22.000)	0.013
ALT [median (IQR), U/L]	16.000 (11.000, 25.475)	16.000 (10.575, 27.000)	15.950 (12.500, 20.550)	0.938
Scr [median (IQR), umol/L]	77.000 (63.350, 95.000)	74.000 (60.000, 88.000)	97.000 (79.000, 120.000)	<0.001
UA [median (IQR), umol/L]	335.000 (266.000, 415.000)	311.000 (250.500, 379.250)	435.000 (368.250, 504.500)	<0.001
BUN [median (IQR), mmol/L]	6.580 (4.400, 9.890)	5.790 (4.087, 8.448)	10.500 (7.225, 12.100)	<0.001
P [median (IQR), mmol/L]	0.930 (0.770, 1.110)	0.940 (0.760, 1.112)	0.920 (0.770, 1.070)	0.893
HB [median (IQR), g/L]	85.000 (68.250, 109.000)	91.000 (74.000, 115.000)	68.500 (61.000, 83.250)	<0.001

### 3.2 The application of 5 ML models

Table 2 presents a comparison between the training and test sets used for ML modeling. The distribution of all variables showed no statistically significant differences between the two sets (all *p* > 0.05). This outcome confirms the successful random partitioning of the dataset, ensuring that baseline characteristics were balanced across both the model development and validation sets. As a result, the data foundation supports the creation of a predictive model with robust generalizability.

**Table 2:** Distributional differences between the training set and test set.

Variables	Training set	Test set	*p*
n	503	215	
Gender, *n* (%)			0.314
Male	400 (79.5)	163 (75.8)	
Female	103 (20.5)	52 (24.2)	
Age, [median (IQR), years]	61.000 (48.000, 73.000)	61.000 (44.000, 73.000)	0.725
Smoking history, *n* (%)			0.1
No	382 (75.9)	176 (81.9)	
Yes	121 (24.1)	39 (18.1)	
Drinking history, *n* (%)			0.126
No	379 (75.3)	174 (80.9)	
Yes	124 (24.7)	41 (19.1)	
BMI [median (IQR), kg/m2]	21.670 (20.450, 24.220)	21.590 (20.470, 24.050)	0.88
FPG [median (IQR), mmol/L]	6.410 (5.070, 8.400)	6.340 (5.020, 8.430)	0.72
LDLC [median (IQR), mmol/L]	1.980 (1.440, 2.525)	1.890 (1.490, 2.525)	0.858
HDLC [median (IQR), mmol/L]	0.860 (0.655, 1.055)	0.860 (0.690, 1.090)	0.4
AST [median (IQR), U/L]	19.000 (15.000, 28.300)	19.400 (15.050, 29.600)	0.531
ALT [median (IQR), U/L]	15.400 (11.000, 25.450)	16.700 (11.300, 25.500)	0.41
Scr [median (IQR), umol/L]	77.000 (64.000, 97.000)	78.000 (63.000, 94.000)	0.862
UA [median (IQR), umol/L]	335.000 (266.000, 416.000)	333.000 (267.000, 413.500)	0.491
BUN [median (IQR), mmol/L]	6.760 (4.300, 10.050)	6.420 (4.665, 9.585)	0.717
P [median (IQR), mmol/L]	0.930 (0.770, 1.105)	0.930 (0.750, 1.110)	0.803
HB [median (IQR), g/L]	84.000 (69.000, 105.500)	89.000 (68.000, 112.500)	0.47

**Figure 1:**
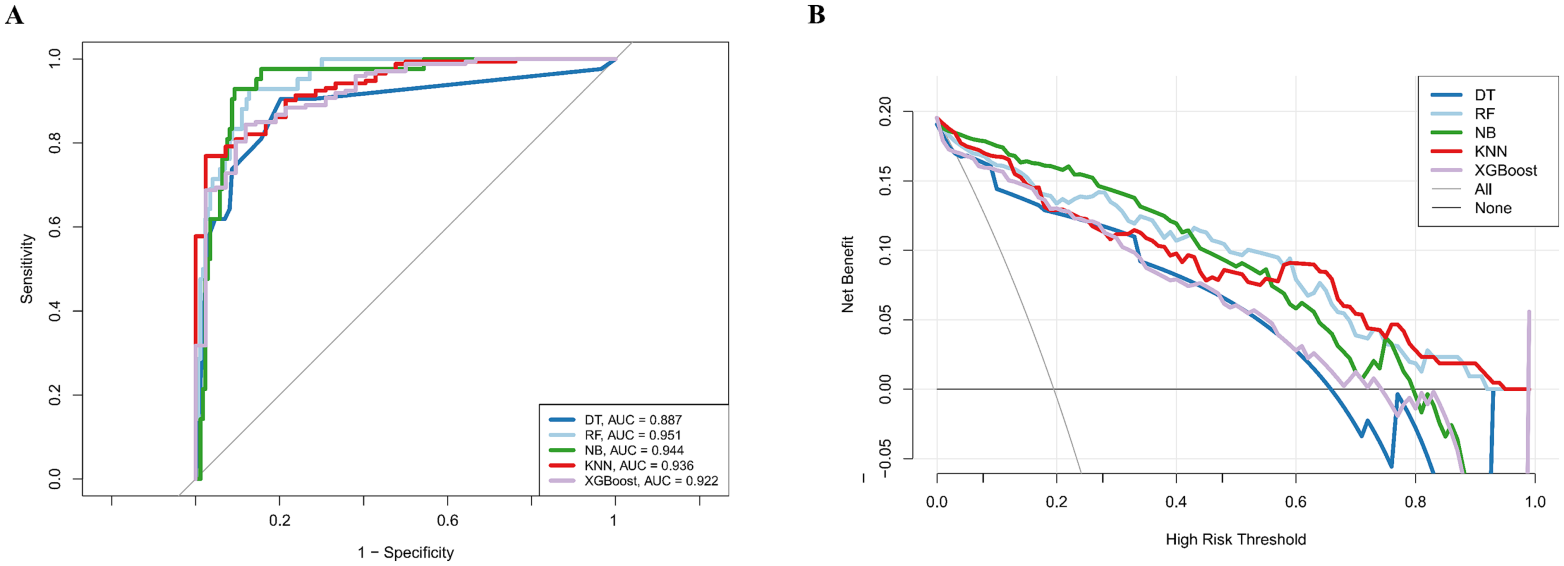
The predictive performance of 5 models on the test set using ROC curves and decision curves. **(A)** ROC curves. **(B)** Decision curves.

Table 3 presents the predictive performance of five ML models on the independent test set. All models demonstrated strong predictive efficacy, with AUC values surpassing 0.887 and accuracy exceeding 0.851, highlighting the overall effectiveness of ML approaches for gout prediction. The RF model achieved the highest performance, with an AUC of 0.951 (95% CI: 0.923–0.979), outperforming the other models. Additionally, the NB model excelled in accuracy (0.918) and specificity (0.908), while maintaining high sensitivity (0.929). The XGBoost and KNN models attained AUC values of 0.922 and 0.936, respectively, demonstrating robust discriminative ability. Despite its relatively simpler architecture, the DT model achieved an AUC of 0.887, with notably high sensitivity (0.905). The ROC curves and decision curves for all five models are presented in Figure 1. Decision curve analysis revealed that all models provided a higher clinical net benefit than the extreme strategies (treat all or treat none) across a range of threshold probabilities. Based on a comprehensive evaluation of all performance metrics, the RF model emerged as the optimal predictive architecture in this study. The multivariable logistic regression model achieved an AUC of 0.898 and an accuracy of 0.822, which were inferior to those of the RF, NB, KNN, and XGBoost models (Supplementary Table 1). This finding demonstrates the superiority of our ML analytical framework over conventional logistic regression. Supplementary Figure 2 shows the calibration curve for the RF model, with predicted probabilities binned into five intervals. The calibration curve consistently fell below the ideal diagonal, indicating a tendency to overestimate observed risks. The Brier score (0.073) and the Hosmer-Lemeshow test (*p* = 0.25) confirmed the model’s high predictive accuracy.

**Table 3:** Performance of five models in the test set.

Metrics	DT	RF	KNN	NB	XGBoost
AUC (95%CI)	0.887 (0.802, 0.953)	0.951 (0.923, 0.979)	0.936 (0.902, 0.969)	0.944 (0.909, 0.978)	0.922 (0.878, 0.965)
Accuracy	0.851	0.901	0.873	0.918	0.862
Sensitivity	0.905	0.929	0.976	0.929	0.881
Specificity	0.798	0.873	0.769	0.908	0.844
PPV	0.521	0.639	0.506	0.709	0.578
NPV	0.972	0.981	0.993	0.981	0.967
Kappa value	0.549	0.684	0.551	0.748	0.605
F1 score	0.661	0.757	0.667	0.804	0.698

### 3.3 SHAP analyses

**Figure 2:**
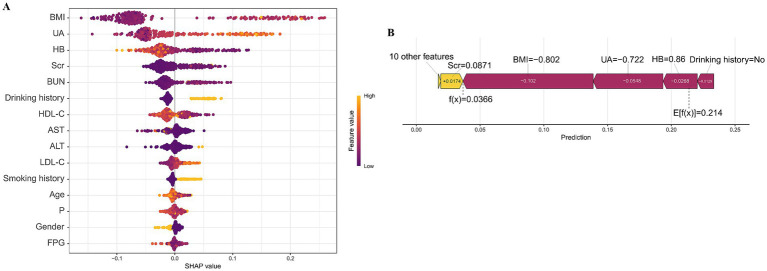
SHAP analysis based on the RF model. **(A)** Beeswarm plot. **(B)** Force plot for the prediction of a random individual.

Figure 2 presents the interpretability analysis of the RF model via SHAP analysis. Panel A (Beeswarm plot of SHAP values) visually summarizes the overall contribution and directional effect of each feature on the model’s prediction of gout risk. Features are ranked in descending order according to their mean absolute SHAP values. BMI emerged as the most influential variable, followed by UA, HB, Scr, BUN, and drinking history. Specifically, elevated levels of BMI, UA, Scr, and BUN were strongly associated with an increased risk of gout, while higher HB levels and the absence of a drinking history were linked to a reduced risk of gout.

Panel B (Individual prediction explanation) further illustrates the contribution of each feature to a specific prediction using a representative sample. The standardized BMI of −0.802 kg/m^2^, standardized UA of −0.722 umol/L, standardized HB of 0.86 g/L, and absence of drinking history exerted negative effects on the prediction. Conversely, standardized Scr of 0.0871 umol/L and other 10 features had positive effects on the prediction. Ultimately, the mean predicted probability increased to 0.214, compared to the base predicted probability of 0.0366.

**Figure 3:**
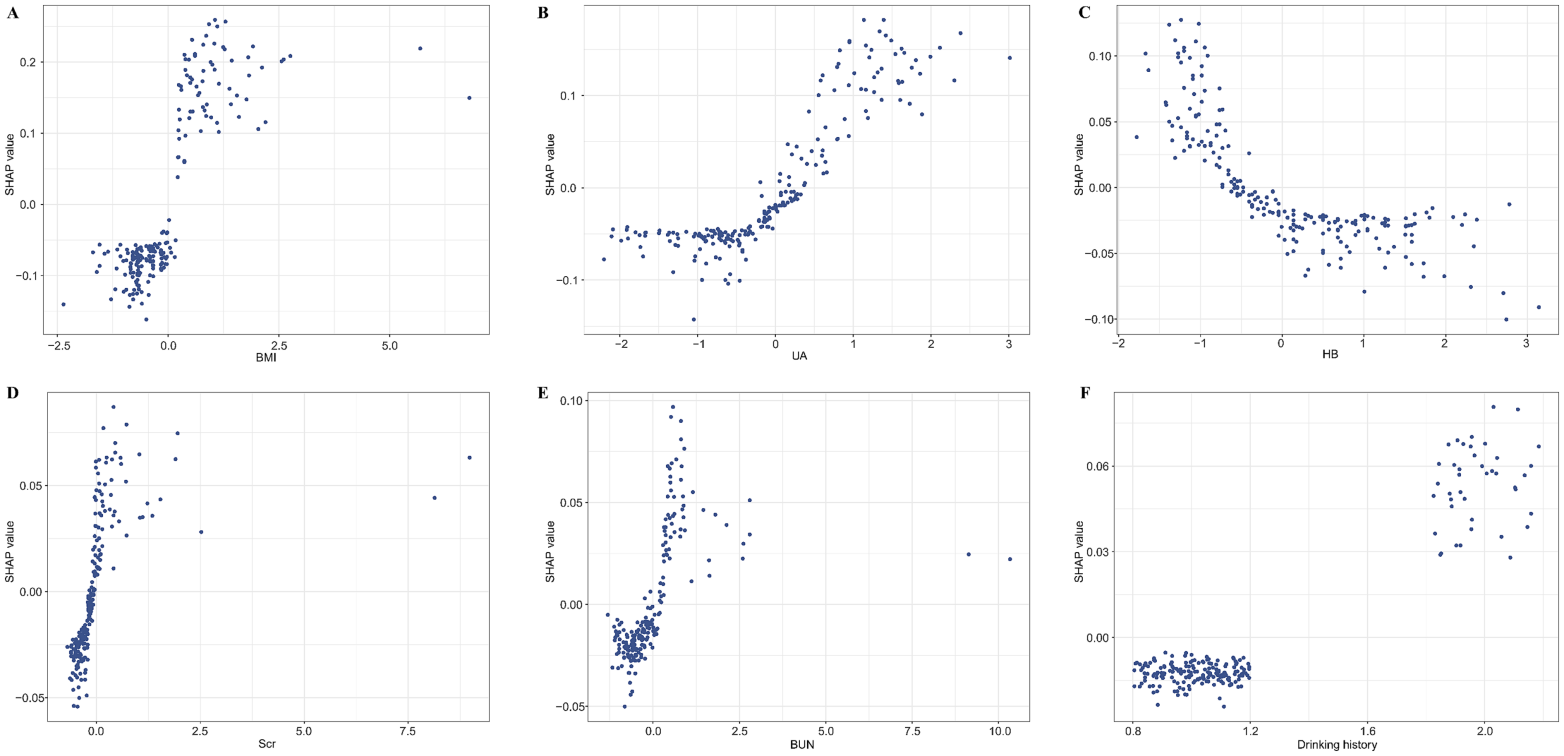
SHAP dependence plots about the correlation of 6 features and gout. **(A)** BMI. **(B)** UA. **(C)** HB. **(D)** Scr. **(E)** BUN. **(F)** Drinking history.

Figure 3 shows the SHAP dependence plots, exploring the non-linear relationships between the six key features and the predicted risk of gout. Higher levels of BMI, UA, Scr, and BUN were linearly associated with an increased risk of gout, while higher HB levels were inversely correlated with gout incidence. These findings suggest that there is no threshold effect for these critical features regarding gout risk.

### 3.4 Shiny web application

The web application is accessible at https://hsh0307.shinyapps.io/45545/ and can predict the risk of gout in UGIB patients. This tool enables clinicians and researchers to input individualized variables and quantify the risk of a specific individual developing gout.

## 4 Discussion

### 4.1 Principal findings and clinical implications

This study is the first to develop and validate an ML model specifically for predicting gout flares during hospitalization in patients with UGIB. The model demonstrated strong discrimination and clinical relevance, providing a valuable tool for the early identification of high-risk patients.

Feature importance analysis consistently identified six key predictors: BMI, Scr, serum UA, history of alcohol consumption, HB, and BUN. These variables reflect the pathophysiological basis of gout flares from diverse perspectives:

Metabolic and Renal Function Indicators (BMI, UA, Scr, BUN): Elevated BMI is often linked to insulin resistance and reduced renal UA excretion (21, 22). Serum UA directly reflects the risk of urate pool saturation (23). Creatinine and BUN are markers of glomerular filtration function. In the context of UGIB, hypovolemia may lead to prerenal acute kidney injury (24), impairing UA excretion and creating a cycle of “renal dysfunction–UA retention,” significantly increasing the risk of gout flares (25, 26). This aligns with established predictors in gout flare rules, such as serum urate levels (27).
History of alcohol consumption: Alcohol is a well-known trigger for acute gout flares as it promotes endogenous UA production and, through its metabolite lactate, competitively inhibits renal tubular UA excretion (27, 28).
HB: As a dynamic marker of UGIB severity, a lower HB level at admission indicates greater blood loss, exacerbating hypovolemia and reducing renal perfusion, which in turn impairs UA excretion. Anemia may also reflect a worse overall clinical condition or underlying chronic disease burden. Predictive models for major gastrointestinal bleeding have also identified HB as a key protective factor, emphasizing its relevance in acute clinical settings (29, 30).

From a pathophysiological standpoint, acute UGIB can directly trigger gout flares. Acute blood loss reduces the effective circulating volume, leading to renal hypoperfusion and a subsequent decrease in glomerular filtration rate. These renal hemodynamic changes critically impair UA excretion, causing a rapid rise in serum urate levels and potentially precipitating an acute flare in susceptible individuals. This mechanistic link highlights the unique vulnerability of UGIB patients to gout flares. Currently, no effective risk-stratification tools exist to predict this complication, hindering early identification and preventive management. Notably, recent research has highlighted PPI use as a potential risk factor for gout flares in UGIB patients, further illustrating the complexity of this clinical scenario.

### 4.2 Comparison with existing literature

Prior research has primarily focused on gout risk in the general population or in patients with chronic kidney disease (31, 32), with limited emphasis on acute gout flares during hospitalization for medical emergencies such as UGIB. A notable study led to the development and validation of the GOUT-36 prediction rule for inpatient gout flares, providing a useful tool for general hospitalized patients with comorbid gout (33, 34). Our study extends this work by specifically targeting the “hospitalization period” in the distinct, high-risk UGIB population, emphasizing the interaction between acute physiological disturbances (e.g., hypovolemia, renal hypoperfusion) and the acute exacerbation of chronic metabolic disorders. This approach enhances clinical timeliness and specificity. Our model demonstrates that combining acute indicators (e.g., HB, BUN) with chronic baseline factors (e.g., BMI, alcohol history, baseline UA) enables a more comprehensive risk assessment tailored to this unique clinical context. Unlike the GOUT-36 rule, which does not incorporate acute dynamic indicators such as HB or BUN, our model includes these UGIB-specific parameters. Moreover, GOUT-36 requires pre-admission serum urate levels and urate-lowering therapy (ULT) history, which may not be readily available or relevant in the acute UGIB setting. Our model, trained specifically on UGIB patients, utilizes routinely available admission laboratory tests, making it more practical for use in emergency settings.

### 4.3 Emerging therapeutic perspectives

The recently published International Clinical Practice Guideline of Chinese Medicine-Gout (SCM73-2024) provides an updated, evidence-based framework that integrates traditional Chinese medicine approaches with contemporary gout management strategies (15). This guideline highlights complementary and alternative medicine (CAM) modalities, such as herbal formulations and acupuncture, as potential adjunctive or alternative treatments for patients with contraindications to conventional therapies. A recent systematic review and meta-analysis by Chen et al. synthesized evidence from multiple randomized controlled trials, showing that CAM interventions—especially Chinese herbal medicine—significantly reduced serum UA levels and improved clinical symptom resolution in gout patients, with favorable safety profiles (35). These findings are particularly relevant to our UGIB cohort, in which standard anti-inflammatory agents (NSAIDs, glucocorticoids) and colchicine may carry heightened risks of gastrointestinal adverse events or drug–drug interactions. While our predictive model does not directly evaluate CAM efficacy, identifying high-risk patients with this tool could enable early consideration of alternative therapeutic strategies when conventional treatments are contraindicated. Future prospective studies should explore whether incorporating CAM interventions into the management of high-risk UGIB patients with impending gout flares can improve outcomes without exacerbating bleeding risks.

### 4.4 Strengths, clinical potential, and limitations

The ML algorithms used in this study (e.g., RF, XGBoost) effectively integrated multidimensional indicators, outperforming traditional logistic regression models, a result consistent with other studies applying advanced algorithms to UGIB prognosis (13, 36). Decision curve analysis confirmed that, within reasonable threshold probabilities, utilizing this model to guide clinical decisions—such as early hydration, urine alkalinization, or prophylactic medication for high-risk patients (37, 38)—could yield significant net clinical benefits. This allows frontline clinicians to quickly assess risk using routinely available parameters early after admission.

This study has several limitations. First, its single-center, retrospective design may be subject to selection bias. To mitigate this, objective inclusion criteria based on electronic medical records were applied to minimize subjective participant selection, and all eligible patients were enrolled consecutively during the study period, avoiding selective sampling. Second, some potential confounders, such as pre-admission ULT and prior joint history—factors included in established prediction rules (39)—may not have been fully captured. However, patients with a history of gout or gout flare within 24 h of admission were excluded. In real-world clinical practice, many UGIB patients with gout are already on ULT.

Third, external validation of the model requires further testing in multicenter, prospective cohorts. Future research could explore incorporating inflammatory markers (40) or imaging features to enhance predictive accuracy.

Fourth, although prior studies have identified PPI use as a potential risk factor for gout flares, medication exposure was not included in our predictor set. PPI use is nearly universal in UGIB patients, limiting its discriminative ability, and the temporal relationship between medication initiation and gout flares is difficult to establish. Future prospective studies should explore the interaction between medication factors and biochemical parameters.

Fifth, although the study period spans from 2012 to 2025, during which clinical practices may have evolved, this temporal variation is unlikely to introduce unmeasured confounding. Our modeling primarily relies on routinely collected demographic characteristics, lifestyle variables, and laboratory measurements, all of which are relatively stable over time and not sensitive to temporal changes. Therefore, the robustness of our analysis remains reliable.

In addition, the use of imbalance sampling strategies, such as synthetic minority over-sampling technique (SMOTE), was considered to address the class imbalance (gout incidence: 22.0%). However, given the potential non-linear relationships between predictors and outcomes, as well as the minority class proportion exceeding the conventional threshold for SMOTE application, the original class distribution was retained to better reflect real-world clinical settings. The strong performance of all models on the test set suggests that severe imbalance-related bias was not present.

## 5 Conclusion

In conclusion, this study developed and validated an ML-based risk prediction model for acute gout flares during hospitalization in UGIB patients. The model demonstrated strong predictive performance and clinical utility. Six key predictors—BMI, Scr, serum UA, alcohol history, HB, and BUN—were identified. These indicators collectively reflect the patient’s metabolic status, renal function, and the severity of acute bleeding. This tool can assist clinicians in the early identification of high-risk patients, enabling targeted monitoring and preventive interventions, ultimately improving in-hospital management and patient outcomes. Furthermore, in light of emerging evidence from the SCM73-2024 guideline and recent meta-analyses on complementary therapies (35), future research should explore whether alternative modalities recommended in the guidelines could benefit UGIB patients at high risk for gout flares who are ineligible for conventional treatment. Prospective studies are needed to further validate and refine this predictive tool.

## Data Availability

The original contributions presented in the study are included in the article/Supplementary material, further inquiries can be directed to the corresponding authors.
