# Supplementary material for: Machine learning-based prediction of gout flares during hospitalization in patients with upper gastrointestinal bleeding: a retrospective cohort study
Source: Front Med (Lausanne). 2026 Jun 10;13:1807548. doi: 10.3389/fmed.2026.1807548 (PMC13290687; doi:10.3389/fmed.2026.1807548)
Supplement: Supplementary file 1 [file Data_Sheet_1.DOCX]

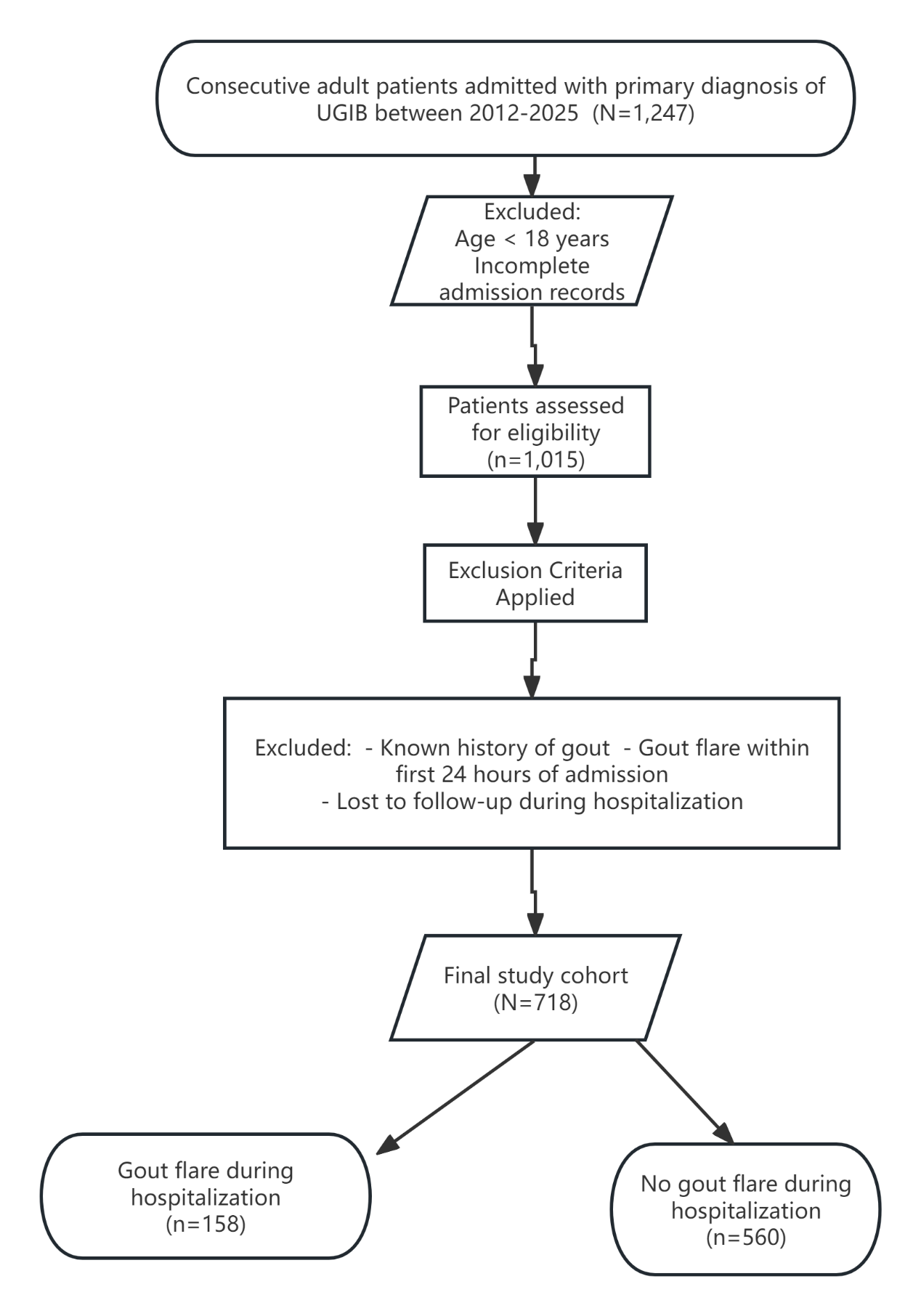


**Supplement Figure 1.** Screening flow of participants.


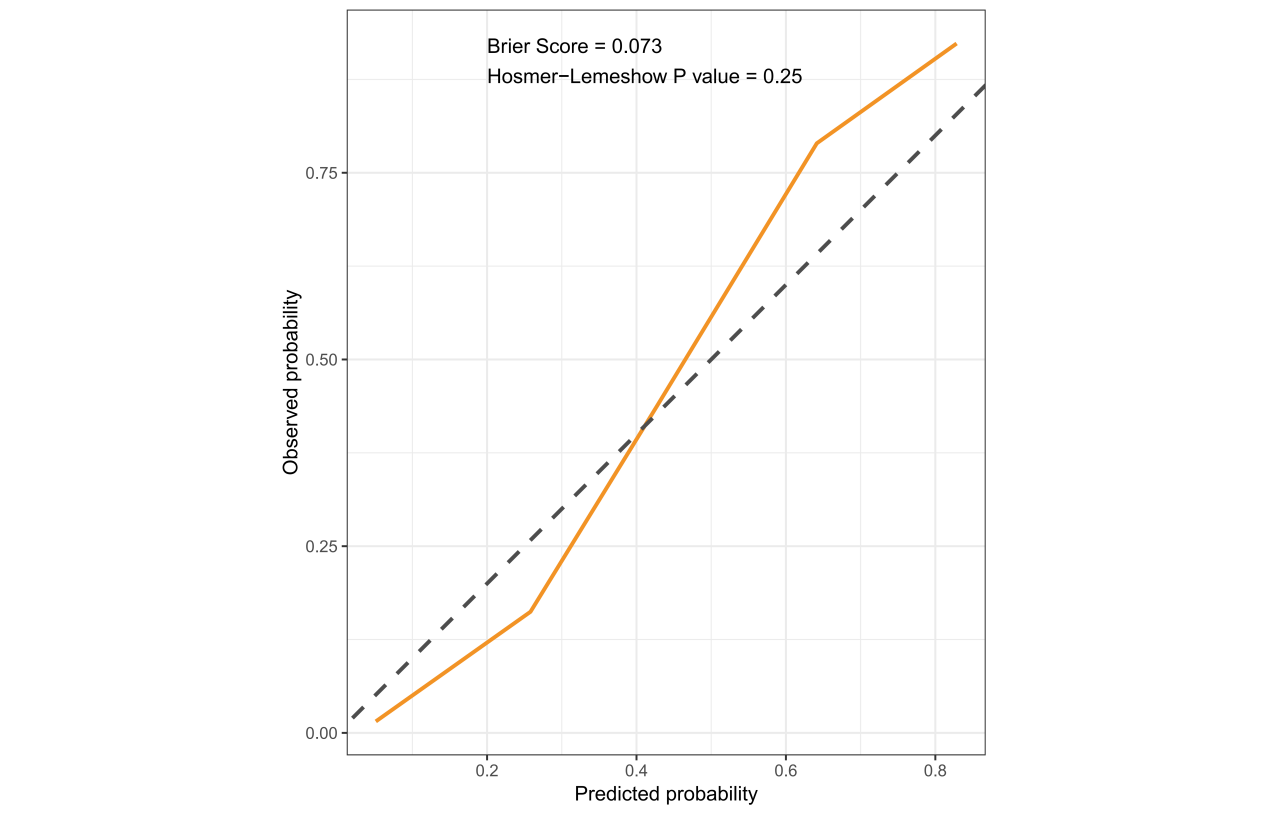


**Supplement Figure 2.** Calibration curve of RF model.

**Supplement Table 1.** The performance of logistic regression model in the test set.

|  | Logistic regression |
| --- | --- |
| AUC (95%CI) | 0.898 (0.849, 0.947) |
| Accuracy | 0.822 |
| Sensitivity | 0.927 |
| Specificity | 0.717 |
| PPV | 0.437 |
| NPV | 0.976 |
| Kappa value | 0.451 |
| F1 score | 0.594 |
